# Microbiological Profile of Organisms Causing Bloodstream Infection in Critically Ill Patients

**DOI:** 10.4021/jocmr1099w

**Published:** 2012-11-11

**Authors:** Jose Orsini, Carlo Mainardi, Eliza Muzylo, Niraj Karki, Nina Cohen, George Sakoulas

**Affiliations:** a Department of Medicine, New York University School of Medicine at Woodhull Medical and Mental Health Center, USA; b Divisions of Pharmacy and Infectious Diseases at Memorial Sloan-Kettering Cancer Center, USA; c Department of Medicine and Division of Infectious Diseases, New York Medical College at Westchester Medical Center, USA

**Keywords:** Bloodstream infection (BSI), Multidrug-resistant (MDR), Extended-spectrum β-lactamase (ESBL), Carbapenem-resistant *Enterobacteriaceae* (CRE), Intensive care units (ICU’s)

## Abstract

**Background:**

Bloodstream infection (BSI) is the most frequent infection in critically ill patients. As BSI’s among patients in intensive care units (ICU’s) are usually secondary to intravascular catheters, they can be caused by both Gram-positive and Gram-negative microorganisms as well as fungi. Infection with multidrug-resistant (MDR) organisms is becoming more common, making the choice of empirical antimicrobial therapy challenging. The objective of this study is to evaluate the spectrum of microorganisms causing BSI’s in a Medical-Surgical Intensive Care Unit (MSICU) and their antimicrobial resistance patterns.

**Methods:**

A prospective observational study among all adult patients with clinical signs of sepsis was conducted in a MSICU of an inner-city hospital in New York City between May 1, 2010 and May 30, 2011.

**Results:**

A total of 722 adult patients with clinical signs of systemic inflammatory response syndrome (SIRS) and/or sepsis were admitted to the MSICU between May 1, 2010 and May 30, 2011. From those patients, 91 (12.6%) had one or more positive blood culture. A 122 isolates were identified: 72 (59%) were Gram-positive bacteria, 38 (31.1%) were Gram-negative organisms, and 12 (9.8%) were fungi. Thirteen (34.2%) Gram-negative organisms and 14 (19.4%) Gram-positive bacteria were classified as MDR.

**Conclusions:**

Antimicrobial resistance, particularly among Gram-negative organisms, continues to increase at a rapid rate, especially in the ICU’s. Coordinated infection control interventions and antimicrobial stewardship policies are warranted in order to slow the emergence of resistance.

## Introduction

Despite advances in therapy and supportive care, BSI continues to be a major cause of morbidity and mortality in hospitalized patients. ICU’s are often the epicenter of these infections, mainly because of its extremely vulnerable population and the increased risk of becoming infected through multiple invasive therapeutic and diagnostic procedures. Accompanying the physiologic stress of infections is the increasingly added burden of MDR that hinders therapy of these infections, with consequential adverse clinical and economic results. The ongoing emergence of resistance in the community and hospitals is a major threat for the public health system [1]. It is becoming increasingly difficult to define the epidemiology of MDR organisms within a specific location or healthcare setting, because of the frequent transfer of patients between acute and long-term care facilities as well as patient migration between different regions. The distinction between nosocomial and community-acquired infections is becoming blurred as well, since community-acquired organisms have become important cause of hospital-acquired infections [2]. Describing the magnitude of the problem with respect to these antimicrobial-resistant pathogens is challenging, because the levels of antimicrobial resistance vary for different types of healthcare facilities and from different geographic areas. Infections with MDR organisms can lead to inadequate or delayed antimicrobial therapy, and are associated with adverse patient’s outcomes [3].

Infection control measures have important implications for daily practice, because the number of patients already colonized or infected with MDR organisms on arrival to the ICU’s is increasing [4]. The objective of this study is to evaluate the spectrum of pathogens causing BSI’s in a MSICU of an inner-city hospital in New York City, as well as their antimicrobial resistance patterns.

## Methods

### Study design and patient population

The study was conducted prospectively in a 346-bed community inner-city hospital located in North Brooklyn, New York, with wards consisting of general internal medicine, general surgery, obstetrics/gynecology, psychiatry, and pediatrics. The total annual number of emergency department visits at our institution is roughly 100,000, with 17,500 admissions per year and an average daily census of about 300 patients. The MSICU in this hospital is comprised of 24 beds. All adult patients admitted to the MSICU between May 1, 2010 and May 30, 2011 were enrolled in the study. The admissions to the MSICU included patients from the emergency department and transfers from the medical and surgical wards. Eligibility criteria included age of 18 years or older, and having at least 2 of the clinical signs or manifestations of SIRS and/or sepsis (defined below). Exclusion criteria were readmission to the MSICU within 30 days, and patients admitted to the unit for short-term post-operative monitoring. Computerized medical records were reviewed and clinical information was abstracted for each patient. Institutional Review Board of the hospital approved the study.

### Definitions

Clinical criteria of SIRS and sepsis were the following: temperature of > 38 °C or < 36 °C, systolic blood pressure of < 90 mmHg, diastolic blood pressure of < 60 mmHg, heart rate of > 90 beats/minute, respiratory rate of > 20 breaths/minute, and white blood cell count of > 12,000/mm^3^ or < 4,000/mm^3^. BSI was defined as infection confirmed by blood culture. MDR in Gram-negative organisms was defined as resistance to at least 1 antimicrobial in 3 or more antimicrobials classes: fluoroquinolones, 3rd generation cephalosporins, aminoglycosides, and carbapenems [5], including organisms harboring Extended-Spectrum β-Lactamases (ESBL’s) [6], and Carbapenem-Resistant *Enterobacteriaceae* (CRE) [7]. Among the Gram-positive organisms, MDR was defined as methicillin resistance in *Staphylococcus aureus* and vancomycin resistance in *Enterococcus spp*. Polymicrobial BSI was described as the recovery of different organisms from one or more blood cultures within the same BSI episode [8].

### Laboratory methods and susceptibility testing

At least one set of blood culture per patient was drawn, and then sent to a reference microbiology laboratory (Kings County Hospital, New York) for processing. The blood culture system Bact-Alert 3D^®^ was used for the recovery of pathogens. Blood culture bottles were incubated for 5 days at 37 °C. Isolates of microorganisms were identified by conventional biochemical and serological methods. A BSI was defined as isolation of at least one positive peripheral blood culture, except cases of infection with coagulase-negative *staphylococci* (CNS), for which isolation of two positive blood cultures was required. Antimicrobial susceptibility testing of isolated pathogens to clinically relevant antimicrobials was performed by using MicroScan panels or the Kirby Bauer diffusion methods, according to the guidelines published by the Clinical and Laboratory Standards Institute (CLSI). Susceptibilities for linezolid, daptomycin, and tigecycline in cases of methicillin-resistant *Staphylococcus aureus* (MRSA) and vancomycin-resistant *Enterococcus* (VRE) were performed as per physician request, and not on routine basis. Gram-negative organisms were tested for ESBL production in cases of isolates with reduced susceptibility to ceftriaxone or ceftazidime. ESBL and carbapenem-resistant organisms were tested for susceptibility to polymyxins as per physician request. Susceptibility testing was not routinely performed for fungal organisms.

## Results

During the 13-month study period, a total of 722 adult patients with clinical signs of SIRS and/or sepsis were admitted to the MSICU. Ninety-one (12.6%) had one or more positive blood culture. 47.2% (43/91) of the isolates were recovered from males. The median age was 64 years (49.4% (45/91) were > 65).

Of 122 isolates recovered, 72 (59%) were Gram-positive bacteria, 38 (31.1%) were Gram-negative organisms, and 12 (9.8%) were fungi. A total of 20 patients (21.9%) had polymicrobial BSI. Among the Gram-positive isolates, the most common organism identified was CNS (28/72, 38.8%), followed by *Staphylococcus aureus* (15/72, 20.8%), *Enterococcus spp* (12/72, 16.6%), and *Streptococcus pneumoniae* (8/72, 11.1%). Seventy-five percent (21/28) of CNS isolates were considered microbiological contaminants and not clinically significant. The most common CNS isolated were: *epidermidis* (14/28, 50%), followed by *hominis* (7/28, 25%), and *haemolyticus* (3/28, 10.7%). All CNS were susceptible to vancomycin, and 75% (21/28) were resistant to methicillin. From the *Staphylococcus aureus* isolates, 9 (60%) were methicillin-susceptible (MSSA), and 6 (40%) were MRSA. Resistance rates for MRSA were as follows: erythromycin, 100% (6/6); clindamycin, 66.6% (4/6); fluoroquinolones, 83.3% (5/6); and sulfamethoxazole, 0% (0/6). The median minimun inhibitory concentration (MIC) for vancomycin in the MRSA group was 2.0 µg/mL.

Among the *Enterococcus spp*, 8 isolates (66.6%) were VRE: 3 isolates (37.5%) were *Enterococcus faecalis* that were susceptible to ampicillin, while 5 (62.5%) were *Enterococcus faecium* resistant to ampicillin; resistance to gentamicin and streptomycin synergy was found in 4 (50%) and 5 (62.5%) isolates, respectively. Susceptibility testing was not performed for linezolid, daptomycin, or tigecycline in cases of MRSA and VRE. All *Streptococcus pneumoniae* isolates were susceptible to penicillin.

The most common Gram-negative bacteria isolated were *Klebsiella spp* (10/38, 26.3%); 8 isolates (80%) were *Klebsiella pneumoniae*. Other relevant Gram-negative organisms were: *Escherichia coli* (8/38, 21%), *Acinetobacter baumannii* (5/38, 13.1%), and *Enterobacter cloacae* (5/38, 13.1%). A total of 13 (34.2%) Gram-negative organisms were identified as MDR, among which 12 (92.3%) produced ESBL’s. The most common ESBL-producers were *Klebsiella pneumoniae* (6/12, 50%), and *Acinetobacter baumannii* (4/12, 33.3%). Carbapenem-resistant phenotype was found in 9 isolates (75%): 5 (55.5%) were *Klebsiella pneumoniae*, and 4 (44.4%) were *Acinetobacter baumannii*. The most common risk factors for MDR infections were diabetes mellitus and malignancy.

Fungal organisms isolated were *Candida glabrata* (4/12, 33.3%), *Candida tropicalis* (4/12, 33.3%), and *Candida albicans* (3/12, 25%). *Aspergillus niger* was identified from one isolate, and was considered as environmental contaminant.

## Discussion

The global escalation in both community- and hospital-acquired antimicrobial-resistant bacteria is increasingly compromising effective antimicrobial therapy, particularly when it comes to empiric antimicrobial selection. The emergence of MDR often is dedicated to excessive use of broad-spectrum antimicrobial agents, since more than 60% of all ICU patients receive antimicrobials during their stay in critical care units [9]. Compared with infections not caused by MDR microorganisms, the additional cost of MDR infections in hospitalized patients have been estimated at $ 6,000 to $ 30,000 per patient [10]. In the battle against MDR, several behavioral changes have been proposed to reduce or to improve antimicrobial therapy. In the ICU’s, strategies such as antimicrobial cycling and de-escalation schemes have been implemented. However, the use of broad-spectrum antimicrobials in critically ill patients is deemed necessary due to small margins for error in choice of therapy, where initial selection of antimicrobials covering offending pathogens is of extreme importance [11]. Lipsitch et al concluded that use of antimicrobials for which resistance is not present will be positively associated at the individual level with carriage of bacteria resistant to another antimicrobial but negatively associated at the population level with the prevalence of resistance to the other antimicrobial [12]. The outcome of BSI’s depends on a number of factors. Mortality seems to be strictly related to the severity of infection, underlying diseases, advanced age, and inadequate antimicrobial therapy.

The epidemiology of microbial pathogens causing BSI’s dramatically changed over years, with a concomitant increase in antimicrobial resistance. A nationwide surveillance study conducted in 49 hospitals in USA showed a large prevalence of Gram-positive bacteria causing BSI’s compared with Gram-negative organisms. However, a trend towards an increasing incidence of Gram-negative organisms causing BSI’s has been observed more recently [13-15]. In our study, there was a slightly higher prevalence of Gram-positive bacteria over Gram-negative organisms causing BSI.

According to data provided by the National Healthcare Safety Network (NHSN), 192 MSICU’s in USA reported 578 catheter-related BSI’s in 2010 [16]. MRSA has emerged as the most common hospital acquired pathogen. By 2003, MRSA represented more than 60% of all *Staphylococcus aureus* isolates in USA ICU’s [13, 17]. From 2005 to 2008, the incidence of invasive MRSA infections declined by 34% compared to baseline rates [18]. Because of about 85% of the infections reported through this system were bloodstream infections, the decreased incidence may be the result of intervention bundles to prevent vascular catheter infections rather than a lower organism burden [19]. Historically, vancomycin has been the cornerstone of treatment for patients with serious MRSA infections. Consequently, vancomycin use has been increasing since the mid 1980’s, resulting in the emergence of MRSA with reduced susceptibility to vancomycin. In patients with *Staphylococcus aureus* bacteremia, higher vancomycin MIC’s have been associated with prolonged bacteremia, increased rate of relapse, prolonged hospital stay, and increased mortality [20]. Vancomycin MIC ‘creep’ (rising of vancomycin MIC’s among vancomycin susceptible isolates) in MRSA continues to be implicated in treatment failures, requiring clinicians to target higher vancomycin trough levels for invasive MRSA disease, and to consider alternative antimicrobial therapy when vancomycin MIC is > 2 µg/mL [21]. As outlined in our report, the median MIC for vancomycin was 2.0 µg/mL. Unfortunately, susceptibility testing for linezolid, daptomycin, or tigecycline was not performed on routine basis. The MRSA rate reported in the present study (40%) is comparable to the USA rate of 52.9% described in the National Nosocomial Infections Surveillance (NNIS) data summary for the period 1992 - 2004 [17]. In a large prospective surveillance study, Zhanel et al reported that 22.3% of all *Staphylococcus aureus* isolates in Canadian ICU’s corresponded to MRSA [22]. Seventy-one percent of bacteremic episodes among patients admitted to Italian ICU’s were caused by MRSA, and many European ICU’s reported that about 9% of the MRSA isolates had a MIC of at least 2.0 µg/mL for vancomycin [23, 24].

CNS is among the most common organisms isolated from blood cultures among patients in the ICU’s. Overall, it was the most commonly isolated organism in this study. In most cases are considered contaminants rather than a cause of true infection. Although distinguishing contaminant from true infection remains difficult without a gold standard, the rate of blood cultures contaminated with CNS in this report was much higher than in other studies [25], but similar to USA rates [17].

The emergence of VRE as an increasingly common nosocomial pathogen has created a formidable challenge for both clinicians and infection control officers since it was first described in 1988 [26]. Hospitalized patients, especially those treated in the ICU’s, are the largest reservoir of VRE. In those patients, colonization occurs predominantly in the gastrointestinal tract facilitating widespread of the organism within hospitals. Vancomycin administration in patients already colonized with VRE significantly increased the risk of prolonged VRE carriage, playing a critical role in the nosocomial epidemiology of this organism [27]. The results of this study showed an overwhelming high rate of VRE (66.6%) among *Enterococcus spp* isolates. In one study, Zhanel et al reported that 6.7% of all *Enterococcus spp* in ICU’s were VRE [22]. Similarly, the prevalence of VRE in European ICU’s appears to be irrelevant, according to data reported by the European Antimicrobial Resistance Surveillance Network [28].

ESBL-producing organisms have been described in USA since the 1980’s and have been associated strongly with nosocomial infections. Carbapenams antimicrobials are considered the first-line therapy for ESBL infections, but resistance to this antimicrobial class is becoming widespread. Since the first case of CRE occurred in North Carolina in 1996 [29], infections due to these organisms have been described in most of the USA. The major carbapenemase in the USA is *Klebsiella pneumoniae* (KPC) [30]. The first reports of KPC isolates started to appear in New York hospitals around 2004. In several reports, the nationwide resistance percentage remained high, which suggests that this phenotype may be more widespread and more common than previously recognized [31, 32]. From 2000 to 2007, the proportion of healthcare-associated *Klebsiella pneumoniae* infections that were due to carbapenem-resistant isolates increased from < 1% to > 8% [33]. Consequently, the number of institutions that reported KPC-producing bacteria recovered from ICU patients increased from 23% to 40% during 2009 - 2010 [34]. Kallen et al reported 15,275 isolates of *Pseudomonas aeruginosa*, *Acinetobacter baumannii*, and *Klebsiella pneumoniae* from 803 hospitals in USA. The proportion of MDR isolates were 10%, 60%, and 15% respectively. Sixty-seven percent of those isolates were linked to infections that occurred in ICU’s. Overall, MDR was most commonly reported from the Northeast region of the USA [35]. In Europe, *Escherichia coli* is the most prevalent organism among infections with ESBL-producing bacteria [36]. Exposure to long-term care facilities and previous exposure to broad-spectrum antimicrobials are risk factors that have been consistently associated with CRE infections. Not surprisingly, patients with carbapenem-resistant infections also have significant comorbidities. Our report showed that 75% of the MDR Gram-negative organisms had carbapenem-resistant phenotype. It was most commonly found in *Klebsiella pneumoniae* and *Acinetobacter baumannii* isolates, similar as in previous studies. However, Bertrand et al reported that North America has the lowest rate of ESBL-producing *Klebsiella pneumoniae* when compared to ICU’s in other continents of the world [37]. Nevertheless, as of 2011, KPC’s have been found in at least 10 countries in four continents with notable outbreaks in Israel and USA [38]. Literature regarding appropriate therapy for CRE infections is limited, as are the options of clinically available antimicrobials. Given the restricted antimicrobial options and the potential for further resistance to develop, most institutions have focused on aggressive infection control policies to limit transmission. In our institution, empiric antimicrobial regimens composed by vancomycin plus a 4th-generation cephalosporin (cefepime), or an anti-pseudomonal penicillin (piperacillin-tazobactam), or a carbapenem (doripenem), with or without a macrolide (azithromycin) or a fluoroquinolone (moxifloxacin) is usually initiated in patients admitted with sepsis with or without septic shock. Surprisingly, all recovered isolates of VRE and CRE were not covered initially by our empiric antimicrobial therapy. Although this report does not address patient outcomes, the suboptimal selection of empiric antimicrobial therapy for those patients likely had adverse impact on survival, and surely in length of stay and hospital costs.

It is also important to address the higher rate (9.8%) of fungemia found in our study, when compared with other reports [39]. *Candida albicans* BSI’s are the most common invasive fungal infections among hospitalized patients. In the USA, is currently the fourth leading cause of nosocomial BSI’s among hospitalized patients and third among ICU patients. The incidence and epidemiology of invasive candidiasis in the ICU’s has undergone considerable change in the past decades. Significant regional and geographic variations exist in the incidence of the different *Candida spp*. Kett et al reported *Candida albicans* as the most common *Candida spp* isolated among patients in ICU’s [13, 39]. In some series, *non-albicans spp* account for nearly half of all *Candida* BSI’s. *Candida glabrata* is generally the second most commonly isolated pathogen in North America [13, 40]. Our results showed slight predominance of *non-albicans spp*. Retrospective cohort studies have been undertaken to estimate mortality attributable to candidemia and report rates ranging from 10% to 49%, with added hospital costs of about $ 40,000 per case [41]. The gold standard diagnostic test for invasive candidiasis has been isolation of the organism by blood culture. Detection of candidemia by blood culture often takes more than 24 hours. For certain *spp*, such as *Candida glabrata*, the time can be even longer, leading to a significant delay in appropriate therapy and higher mortality [42, 43]. None of the patients included in this study were receiving empiric antifungal therapy at the time the fungal organisms were isolated.

There are several limitations in this study: 1) MRSA and VRE isolates were not routinely tested for susceptibility to linezolid, daptomycin, or tigecycline; 2) ESBL and CRE isolates were not routinely tested for susceptibility to polymyxins; 3) our institution did not have an antimicrobial stewardship program developed at the time of the study; 4) the practice of surveillance cultures among critically ill patients has not been consistently followed in our institution; 5) we did not know the timing from admission to the ICU to when the blood cultures became positive, so it was difficult to differentiate between community-acquired and hospital-acquired BSI’s; and 6) lack of microbiology laboratory within the facility, so our samples have to be process in another institution. It is difficult to quantify the burden of disease, expressed as increased mortality or prolonged length of stays due to infections caused by these antimicrobial-resistant organisms. Routine surveillance cultures to steer empiric antimicrobial therapy may result in higher rates of initial appropriate therapy while including a substantial potential in the savings of last-line antimicrobial agents [44]. Specific impact of preventive measures certainly depends on the local epidemiology and resistance levels.

This study, along with previous studies, highlights that antimicrobial resistance is high in patients admitted to ICU’s. Infection control measures have important implications for daily practice, because more patients are already colonized with MDR organisms on arrival to ICU. Therefore, broad empiric antimicrobial coverage may be needed in several patients admitted to these units. In the absence of new antimicrobials, prevention of infections and optimizing adherence to universal infection control measures, along with restriction of antimicrobials consumption by a sensible hospital drug policy such as antimicrobial stewardships and promotion of a rational use of antibiotics, should stop or decreased the rising of MDR.

## References

[1] Brusselaers N, Vogelaers D, Blot S (2011). The rising problem of antimicrobial resistance in the intensive care unit. Ann Intensive Care.

[2] Prabaker K, Weinstein RA (2011). Trends in antimicrobial resistance in intensive care units in the United States. Curr Opin Crit Care.

[3] Anderson DJ, Engemann JJ, Harrell LJ, Carmeli Y, Reller LB, Kaye KS (2006). Predictors of mortality in patients with bloodstream infection due to ceftazidime-resistant Klebsiella pneumoniae. Antimicrob Agents Chemother.

[4] Vogelaers D, De Bels D, Foret F, Cran S, Gilbert E, Schoonheydt K, Blot S (2010). Patterns of antimicrobial therapy in severe nosocomial infections: empiric choices, proportion of appropriate therapy, and adaptation rates--a multicentre, observational survey in critically ill patients. Int J Antimicrob Agents.

[5] Magiorakos AP, Srinivasan A, Carey RB, Carmeli Y, Falagas ME, Giske CG, Harbarth S (2012). Multidrug-resistant, extensively drug-resistant and pandrug-resistant bacteria: an international expert proposal for interim standard definitions for acquired resistance. Clin Microbiol Infect.

[6] Nogueira-Miranda Kda S, Palmeiro JK, Conte D, Maia FV, Reason IT, Monteiro CL, Dalla-Costa LM (2012). Detection of extended-spectrum beta-lactamase in Enterobacter spp.--evaluation of six phenotypic tests. Microb Drug Resist.

[7] Cuzon G, Naas T, Nordmann P (2010). [KPC carbapenemases: what is at stake in clinical microbiology?]. Pathol Biol (Pairs).

[8] Luzzaro F, Ortisi G, Larosa M, Drago M, Brigante G, Gesu G (2011). Prevalence and epidemiology of microbial pathogens causing bloodstream infections: results of the OASIS multicenter study. Diagn Microbiol Infect Dis.

[9] Borg MA (2003). Bed occupancy and overcrowding as determinant factors in the incidence of MRSA infections within general ward settings. J Hosp Infect.

[10] Cosgrove SE (2006). The relationship between antimicrobial resistance and patient outcomes: mortality, length of hospital stay, and health care costs. Clin Infect Dis.

[11] Prowle JR, Heenen S, Singer M (2011). Infection in the critically ill--questions we should be asking. J Antimicrob Chemother.

[12] Lipsitch M, Bergstrom CT, Levin BR (2000). The epidemiology of antibiotic resistance in hospitals: paradoxes and prescriptions. Proc Natl Acad Sci U S A.

[13] Wisplinghoff H, Bischoff T, Tallent SM, Seifert H, Wenzel RP, Edmond MB (2004). Nosocomial bloodstream infections in US hospitals: analysis of 24,179 cases from a prospective nationwide surveillance study. Clin Infect Dis.

[14] Munoz P, Cruz AF, Rodriguez-Creixems M, Bouza E (2008). Gram-negative bloodstream infections. Int J Antimicrob Agents.

[15] Blot S, Cankurtaran M, Petrovic M, Vandijck D, Lizy C, Decruyenaere J, Danneels C (2009). Epidemiology and outcome of nosocomial bloodstream infection in elderly critically ill patients: a comparison between middle-aged, old, and very old patients. Crit Care Med.

[16] Dudeck MA, Horan TC, Peterson KD, Allen-Bridson K, Morrell G, Pollock DA, Edwards JR (2011). National Healthcare Safety Network (NHSN) Report, data summary for 2010, device-associated module. Am J Infect Control.

[17] (2004). National Nosocomial Infections Surveillance (NNIS) System Report, data summary from January 1992 through June 2004, issued October 2004. Am J Infect Control.

[18] Kallen AJ, Mu Y, Bulens S, Reingold A, Petit S, Gershman K, Ray SM (2010). Health care-associated invasive MRSA infections, 2005-2008. JAMA.

[19] O'Grady NP, Alexander M, Dellinger EP, Gerberding JL, Heard SO, Maki DG, Masur H (2002). Guidelines for the prevention of intravascular catheter-related infections. Centers for Disease Control and Prevention. MMWR Recomm Rep.

[20] Tenover FC, Moellering RC, Jr (2007). The rationale for revising the Clinical and Laboratory Standards Institute vancomycin minimal inhibitory concentration interpretive criteria for Staphylococcus aureus. Clin Infect Dis.

[21] Liu C, Bayer A, Cosgrove SE, Daum RS, Fridkin SK, Gorwitz RJ, Kaplan SL (2011). Clinical practice guidelines by the infectious diseases society of america for the treatment of methicillin-resistant Staphylococcus aureus infections in adults and children. Clin Infect Dis.

[22] Zhanel GG, DeCorby M, Laing N, Weshnoweski B, Vashisht R, Tailor F, Nichol KA (2008). Antimicrobial-resistant pathogens in intensive care units in Canada: results of the Canadian National Intensive Care Unit (CAN-ICU) study, 2005-2006. Antimicrob Agents Chemother.

[23] Malacarne P, Boccalatte D, Acquarolo A, Agostini F, Anghileri A, Giardino M, Giudici D (2010). Epidemiology of nosocomial infection in 125 Italian intensive care units. Minerva Anestesiol.

[24] Hawser SP, Bouchillon SK, Hoban DJ, Dowzicky M, Babinchak T (2011). Rising incidence of Staphylococcus aureus with reduced susceptibility to vancomycin and susceptibility to antibiotics: a global analysis 2004-2009. Int J Antimicrob Agents.

[25] van der Heijden YF, Miller G, Wright PW, Shepherd BE, Daniels TL, Talbot TR (2011). Clinical impact of blood cultures contaminated with coagulase-negative staphylococci at an academic medical center. Infect Control Hosp Epidemiol.

[26] (1993). Nosocomial enterococci resistant to vancomycin--United States, 1989-1993. MMWR Morb Mortal Wkly Rep.

[27] Yoon YK, Lee SE, Lee J, Kim HJ, Kim JY, Park DW, Sohn JW (2011). Risk factors for prolonged carriage of vancomycin-resistant Enterococcus faecium among patients in intensive care units: a case-control study. J Antimicrob Chemother.

[28] (2010). European Centre for Disease Prevention and Control. Annual epidemiological report on communicable diseases in Europe 2010. Stockholm:ECDC.

[29] Yigit H, Queenan AM, Anderson GJ, Domenech-Sanchez A, Biddle JW, Steward CD, Alberti S (2001). Novel carbapenem-hydrolyzing beta-lactamase, KPC-1, from a carbapenem-resistant strain of Klebsiella pneumoniae. Antimicrob Agents Chemother.

[30] Kitchel B, Rasheed JK, Patel JB, Srinivasan A, Navon-Venezia S, Carmeli Y, Brolund A (2009). Molecular epidemiology of KPC-producing Klebsiella pneumoniae isolates in the United States: clonal expansion of multilocus sequence type 258. Antimicrob Agents Chemother.

[31] Bradford PA, Bratu S, Urban C, Visalli M, Mariano N, Landman D, Rahal JJ (2004). Emergence of carbapenem-resistant Klebsiella species possessing the class A carbapenem-hydrolyzing KPC-2 and inhibitor-resistant TEM-30 beta-lactamases in New York City. Clin Infect Dis.

[32] Hidron AI, Edwards JR, Patel J, Horan TC, Sievert DM, Pollock DA, Fridkin SK (2008). NHSN annual update: antimicrobial-resistant pathogens associated with healthcare-associated infections: annual summary of data reported to the National Healthcare Safety Network at the Centers for Disease Control and Prevention, 2006-2007. Infect Control Hosp Epidemiol.

[33] (2009). Guidance for control of infections with carbapenem-resistant or carbapenemase-producing Enterobacteriaceae in acute care facilities. MMWR Morb Mortal Wkly Rep.

[34] Nelson SR, Hayden MK, Vernon MO, Won S, Heiman K, Weinstein R Increasing burden of carbapenem-resistant Enterobacteriaceae (CRE) in the Chicago metropolitan area: results of two surveys of infection preventionists [Abstract 360]. In proceedings of the 48th Annual Meeting of the Infectious Diseases Society of America 21-24 October 2010; Alexandria, VA: Infectious Diseases Society of America 2010.

[35] Kallen AJ, Hidron AI, Patel J, Srinivasan A (2010). Multidrug resistance among gram-negative pathogens that caused healthcare-associated infections reported to the National Healthcare Safety Network, 2006-2008. Infect Control Hosp Epidemiol.

[36] Meyer E, Schwab F, Schroeren-Boersch B, Gastmeier P (2010). Dramatic increase of third-generation cephalosporin-resistant E. coli in German intensive care units: secular trends in antibiotic drug use and bacterial resistance, 2001 to 2008. Crit Care.

[37] Bertrand X, Dowzicky MJ (2012). Antimicrobial susceptibility among gram-negative isolates collected from intensive care units in North America, Europe, the Asia-Pacific Rim, Latin America, the Middle East, and Africa between 2004 and 2009 as part of the Tigecycline Evaluation and Surveillance Trial. Clin Ther.

[38] Grundmann H, Livermore DM, Giske CG, Canton R, Rossolini GM, Campos J, Vatopoulos A (2010). Carbapenem-non-susceptible Enterobacteriaceae in Europe: conclusions from a meeting of national experts. Euro Surveill.

[39] Kett DH, Azoulay E, Echeverria PM, Vincent JL (2011). Candida bloodstream infections in intensive care units: analysis of the extended prevalence of infection in intensive care unit study. Crit Care Med.

[40] Horn DL, Fishman JA, Steinbach WJ, Anaissie EJ, Marr KA, Olyaei AJ, Pfaller MA (2007). Presentation of the PATH Alliance registry for prospective data collection and analysis of the epidemiology, therapy, and outcomes of invasive fungal infections. Diagn Microbiol Infect Dis.

[41] Morgan J, Meltzer MI, Plikaytis BD, Sofair AN, Huie-White S, Wilcox S, Harrison LH (2005). Excess mortality, hospital stay, and cost due to candidemia: a case-control study using data from population-based candidemia surveillance. Infect Control Hosp Epidemiol.

[42] Fernandez J, Erstad BL, Petty W, Nix DE (2009). Time to positive culture and identification for Candida blood stream infections. Diagn Microbiol Infect Dis.

[43] Lepak A, Andes D (2011). Fungal sepsis: optimizing antifungal therapy in the critical care setting. Crit Care Clin.

[44] Blot S, Depuydt P, Vogelaers D (2008). Maximizing rates of empiric appropriate antibiotic therapy with minimized use of broad-spectrum agents: are surveillance cultures the key?. Intensive Care Med.

